# Chronic Bilateral Anterior Shoulder Fracture-Dislocation Following Febrile Seizure After COVID-19 Vaccine: A Case Report

**DOI:** 10.7759/cureus.35391

**Published:** 2023-02-24

**Authors:** Batool AlAskar, Abdulaziz Alqahtani, Sultan Alsuhaim

**Affiliations:** 1 Department of Orthopaedic Surgery, Prince Sultan Military Medical City, Riyadh, SAU

**Keywords:** orthopedic sports surgery, missed bilateral anterior shoulder dislocation, shoulder dislocation, febrile seizure, orthopedic procedure

## Abstract

Although the shoulder is one of the most commonly dislocated joints in the body, bilateral gleno-humeral joint dislocation is considered rare. Due to its complexity and paucity of cases reported in the literature, it represents both a diagnostic and therapeutic challenge. We report a rare case of an adolescent boy who suffered chronic bilateral anterior shoulder dislocations with proximal humerus fracture and Hill-Sachs lesion after febrile seizure following COVID-19 vaccination. An 18-year-old male presented with bilateral proximal humerus fracture with anterior shoulder dislocation following a first-time seizure. He was managed with a bilateral Latarjet procedure and proximal humerus interlocking osteosynthesis (PHILOS) on the left side, and the right-side fracture was fixed with two 3.5 mm cannulated screws. After one year, the patient had a somewhat satisfactory outcome with a DASH (disabilities of the arm, shoulder, and hand) score of 31.8. Bilateral anterior shoulder dislocation with associated proximal humerus fracture remains one of the rare orthopedic injuries. Recurrent shoulder dislocations lead to chronic glenoid bone loss, which needs fixation along with fracture.

## Introduction

Seizures and epilepsy carry a significant burden due to their unpredictable course and associated injuries. Seizure-related injuries are due to the forceful contraction of one muscle group during the tonic phase of the seizure, which creates enough force to dislocate the shoulder joint [1]. Shoulder dislocations usually occur in the context of first-time seizures. Although the shoulder is the most commonly dislocated joint in the body, bilateral gleno-humeral joint dislocation is considered rare [2]. Bilateral shoulder dislocations are almost always posterior with only a few reported bilateral anterior dislocations [3,4]. Having bilateral anterior shoulder dislocations with associated proximal humerus fractures is exceedingly rare [4]. Due to its complexity and paucity of cases reported in the literature, it represents both a diagnostic and therapeutic challenge. We thus report a rare case of an adolescent boy who suffered chronic bilateral anterior shoulder dislocations with proximal humerus fracture and Hill-Sachs lesion after a febrile seizure following COVID-19 vaccination and was treated with open reduction and internal fixation and Latarjet procedure.

## Case presentation

**Figure 1:**
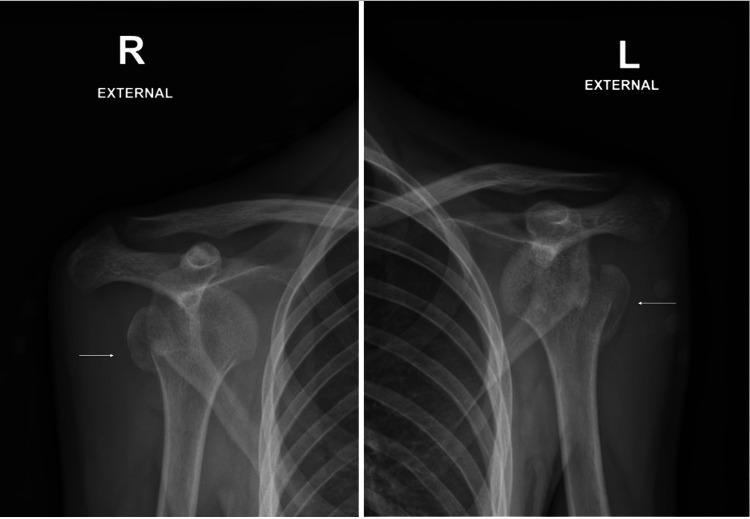
Bilateral shoulder x-rays showing anterior shoulder dislocation with proximal humerus fracture

An 18-year-old boy not known to have any medical illness had presented to receive the COVID-19 vaccine at one of the major vaccination centers in the Northern region of Saudi Arabia. Shortly thereafter, he developed generalized tonic-clonic seizure. He was immediately transferred to one of the hospitals where he spent three months in the intensive care unit (ICU). After medical stabilization, he presented to the orthopedic department with both shoulders abducted and externally rotated and elbow flexed. He had a loss of range of motion in both shoulders in all planes. No peripheral neurological deficit was noted. Immediate radiographs demonstrated bilateral anterior shoulder dislocations with associated proximal humerus fractures (Figure 1). 

**Figure 2:**
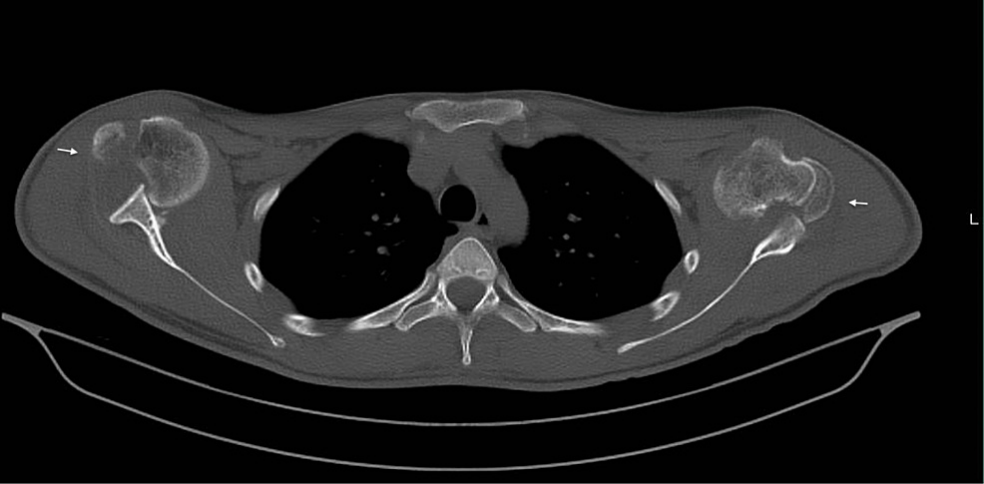
CT scan showing bilateral anterior shoulder dislocation with proximal humerus fractures and Hill-Sachs lesions CT, computed tomography.

Computed tomography (CT) was requested, which revealed bilateral anterior shoulder dislocations associated with greater tuberosity fractures and Hill-Sachs lesions (Figure 2).

**Figure 3:**
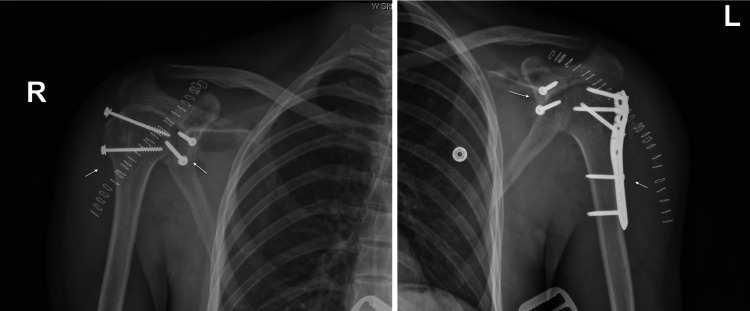
Bilateral shoulder x-rays taken after open reduction of the fractures and internal fixation and Latarjet

There was no significant glenoid bone loss. It was then decided to take the patient for bilateral open reduction of dislocations with open reduction of the fractures and internal fixation and the Latarjet procedure. Both shoulders were done in the same sitting. The deltopectoral approach was utilized; the left-side fracture was fixed using the proximal humerus interlocking osteosynthesis (PHILOS) plating and the right-side fracture was fixed with two 3.5 mm cannulated screws (Figure 3).

Although reduction was somewhat difficult, intraoperative examination revealed stable shoulders with an almost full range of motion. The early postoperative period was uneventful. The shoulders were initially immobilized for the first four weeks with only passive movements allowed at first. Following this period, active exercises were started. The patient achieved flexion: 110-120, abduction: 110, internal rotation: 50-60, and external rotation: 40-50. During the follow-up visits, stability, pain, range of motion, and coracoid healing were regularly checked. Although the patient did not report any pain, he developed persistent stiffness and reduced range of motion in both shoulders due to the chronicity of his shoulder fracture dislocation and noncompliance with physiotherapy, as the patient was from a rural area where physical therapy compliance and efficiency could not be guaranteed. Moreover, stiffness remained the same during one-year visit. At the one-year follow-up, the calculated DASH (disabilities of the arm, shoulder, and hand) score was 31.8.

## Discussion

The first described case of bilateral anterior shoulder dislocation was in 1902 by Myenter after a camphor overdose [5,6]. This was followed by another case described by Sargent secondary to muscle wasting [6]. Ever since, bilateral anterior shoulder dislocation has been described but only rarely. This scarcity may be attributed to the mechanism necessary to produce this injury pattern. The usual mechanism of bilateral dislocation almost always leads to posterior dislocation owing to the relatively weak external rotators overcome by the more powerful internal rotators, with the resultant adduction and internal rotation [7]. Several mechanisms were proposed that can lead to bilateral anterior fracture dislocation. These include forceful bilateral traction, abrupt muscular contractures, or bilateral deceleration forces related o trauma [7,8]. Fractures usually result from direct trauma when the shoulder is beaten against a hard surface as usually happens in the cases of seizures [9,10]. Anterior dislocation is most often associated with two-part proximal humerus fractures [8,9]. Furthermore, greater tuberosity fractures were reported in about 15% of anterior dislocations as demonstrated in this case [8]. Although there is no real consensus on how to treat these rare complex injuries, there are a few factors to consider. These include the patient’s age, level of function, dislocation time, the presence of Hill Sachs and glenoid bone loss, and the surgeon’s preference [9,11]. This case presented a unique challenge as the patient presented late, making closed reduction particularly difficult due to the risk of causing iatrogenic fractures and the risk of iatrogenic neurovascular damage. Chronic shoulder dislocations are particularly difficult to reduce by closed methods due to soft tissue contractures, fibrous tissue in the glenoid cavity, and retracted rotator cuff muscles [12]. However, Deepak et al. still recommend closed reduction even in chronic cases [9]. In addition, these overlooked cases generally tend to have major bony defects due to the continuous motion of the dislocated humeral head against the anterior border of the glenoid. Other possible complications following this injury pattern can include instability, re-dislocation, non-union, malunion, and Bankart lesion. Although our patient did not have significant glenoid bone loss, the senior author still decided to perform Latarjet. First, the patient had a chronic dislocation, which led to soft tissue imbalance with thinning and lengthening of the musculotendinous structure around the shoulder joint, putting him at a high risk of re-dislocation. In addition, he had a significant Hill-Sachs lesion. Latarjet remains the mainstay in most cases as it provides greater stability through its “triple effect” as well as better surgeon familiarity. Poggetti et al. reported excellent results at one year following open reduction and Latarjet procedure in a 28-year-old lady with neglected bilateral anterior shoulder dislocations [11]. As compared to the previous study, our patient achieved less shoulder range of motion and a much higher DASH score. In Rai et al.'s prospective study, seven patients with chronic dislocation were managed with open reduction, Latarjet, and capsulolabral repair. They reported significant improvement in patient's pain relief and functional status [13]. Lubis et al. managed a case of chronic anterior shoulder dislocation with open reduction and Latarjet procedure. The authors concluded that coracoid osteotomy prevents re-dislocation and enhances the functional status of the shoulder joint [14]. Open reduction along with Latarjet is further supported by the case described by Anurag et al. where they performed Latarjet for chronic bony Bankart in a 48-year-old lady with bilateral proximal humerus fracture and anterior dislocation following a seizure attack [15]. 

## Conclusions

Bilateral anterior shoulder dislocation with associated proximal humerus fracture remains one of the rare orthopedic injuries. Delayed presentations along with proximal humerus bone impaction and glenoid defect add to the rarity and complexity of such injury patterns. We believe that our case is an important addition to the literature on bilateral anterior shoulder dislocations that can help guide treatment in similar cases.
